# New York Academy of Medicine.—Section on Orthopedic Surgery

**Published:** 1890-06

**Authors:** 


### Society Proceedings.

### NEW YORK ACADEMY OF MEDICINE.—SECTION ON ORTHOPEDIC SURGERY.

STATED MEETING, APRIL 18, 1890.

V. P. GIBNEY, M. D., Chairman.

### HEMATOMA OF THE STERNO-CLEIDO MASTOID MUSCLE.

Dr. A. B. JUDSON presented a patient, four and a half weeks old, who had been referred to him as a case of congenital torticollis. There was a long fusiform tumor in the course of the muscle, the hardness of which suggested a short and fibrous sterno-cleido mastoid. There was, however, but little shortening, and no wry-neck. The condition was supposed to be the result of injury to the muscle in parturition. Dr. B. E. Hadra, of Texas, had reported two cases which had been relieved by tenotomy, and Dr. F. D. Brooks, of New Hampshire, had followed with a report of three cases, which had recovered by expectant treatment, or the use of friction and local applications. In the present case a favorable prognosis had been given without special treatment.

### A NEW BED FOR USE IN HIP DISEASE.

Dr. A. M. PHELPS presented a little girl with hip disease, who had been treated on an improved surgical bed, which was also exhibited to the Section. When she came under his care, there was flexion nearly to a right-angle, adduction, sinuses and an abscess, and the liver was already enlarged. His improved bed consisted of the ordinary iron bedstead found in hospitals, to which was added a convenient arrangement for the application of traction. The iron bed-posts at the foot of the bed were continued upwards much higher than those at the head. An iron cross-bar slid up and down on these foot-posts, and could be fastened at any height, so as to make traction at any angle desired. This cross-bar carried a pulley, which could be adjusted laterally so as to make traction directly in the line of deformity. The side-bar of the bedstead was also fitted with an adjustable pulley for the purpose of making lateral traction. This apparatus cost about five dollars, and could be supplied by Reynders, either with or without the bedstead.

The patient whom he exhibited had been treated by traction in this bed; but this was not sufficient to overcome the deformity. Under chloroform, the tensor vagina femoris and fascia lata, the adductors longus and magnus, and the contracted anterior border of the glutei muscles and the rectus femoris, were divided. Traction with a weight of eight pounds was then applied in the line of the deformity, and a force of two pounds at right-angles to this. After two months, the deformity had been for the most part reduced, but his splint, with crutches and a high shoe, were then applied to prevent relapse, and they would be continued until the case was cured.

Dr. R. H. Sayre presented

### A CAST OF CONGENITAL LOCK–JAW.

No definite history could be obtained concerning this boy, except that he was five years of age, and that nothing unusual had been noticed about the jaw until a short time ago. The boy was quite intelligent, and no other joints were affected. The jaw appeared to be subluxated backward, and the deformity was presumably congenital. The recession of the jaw, and the apparent atrophy on both sides, added to the interest of the case. Dr. Sayre said that before adopting any operative measures, he would attempt to relieve the case by stretching; and for this purpose would employ a wedge-shaped instrument devised by Dr. L. W. Hubbard, and presented last year before the Society of the Alumni of Bellevue Hospital. It consisted of two plates of steel, fastened together by a separable hinge, and capable of being separated at the other end by turning a screw. Having partly separated the jaws of the instrument, a cork could be inserted between the plates near the hinge, and the action of the screw reversed when the instrument would exert considerable pressure on the molar teeth.

Dr. R. W. Townsend presented two cases of

### RACHITIC POSTERIOR CURVATURE OF TIBIÆ.

He said that the dispensary records showed that about two years ago there was a well-marked knock-knee and rachitis in one case, who returned last week with the present peculiar condition of the tibia. Since then, the other case, with a similar deformity, had come under observation. The latter case presented an increased growth of one portion of the tibia, amounting almost to an exostosis. It also showed a well-marked "rachitic rosary." Macewen had called especial attention to these secondary bone formations on the inner side of the knee in cases of knock-knee. The posterior curves of the tibia were rarely seen, these being the only cases met with during the past two years at the Hospital for Ruptured and Crippled.

Dr. S. KETCH reported a case of

### RHEUMATIC (?) ARTHRITIS OF KNEE.

On July 3, 1888, he was asked to see the following case in consultation with Drs. Dawrence Johnson and N. J. Hepburn:

E. S., single; twenty-two years of age; having a good family history, had been perfectly well up to the present illness, and denied having had any venereal disease; an examination of the urethra failed to show the presence of a urethritis. Early in May, 1888, he had a slight attack of what was considered to be rheumatism in the left elbow and right thumb, which left these joints in a few days, and lodged in the right knee. No other joints became involved; but he grew steadily worse under treatment for rheumatism, and emaciated rapidly after the involvement of the knee. When first seen by Dr. Ketch, he presented the facies of extreme suffering; the knee-joint was flexed beyond ninety degrees; and was very much swollen and excessively tender; there was manifest atrophy of the thigh and calf; pulse 120; temperature, 103.5 degrees F. He had had no chill. Anodynes were constantly required, and his general health was failing rapidly. The urine was abundant, and was free from albumen. Urates were in excess. The acute symptoms continuing unabated after the constant application of ice, and the administration of morphine and the salicylate of soda for several days, the patient was etherized on July 12th, and the knee straightened with the exercise of as little force as possible. Adhesive plasters were applied from below the knee to above the malleoli, and plaster-of-Paris over this with reinforcements by steel bars, the joint being left exposed. The limb was elevated, ice bags applied to the knee, and traction made in a straight line by a weight of ten pounds. This was followed by speedy relief, and a reduction of the temperature to 100 degrees F. On the following day, the swelling had greatly increased, but the limb could be handled quite freely. The joint was firmly bandaged, and the ice continued. On July 16th, Dr. Gibney saw the patient, and advised a continuance of the treatment regardless of the swelling. The patient did not then require anodynes; appetite was improving; and the temperature had fallen to ninety-nine degrees F. Ice bags were continued during the month of August, and the local tenderness diminished more rapidly than the pain on motion. When the splint was removed early in October, there was scarcely any motion at the articulation, and the joint could be freely handled without complaint. A retention splint was applied, and the patient allowed to go about on crutches. In April, 1889, the anchylosis was complete and he was enabled to return to work. He could at present walk long distances without fatigue, and his general condition was good. The chief points of interest in the case were regarding the etiology and the treatment. He believed that there were cases of rheumatism like this one, in which the rheumatic process was modified or entirely changed in character. The presence of a poison in the system was undoubted; but it was remarkable that it should have been so mild at the time the elbow and thumb were attacked; and then have become so concentrated in the knee-joint as to practically destroy it. Rheumatoid arthritis was usually a chronic process involving numerous joints, and eventually crippling them; but such a process was not found in the present instance. The subject of treatment was important as bearing on the treatment of joints affected with rheumatism; and he was positive that his case would have resulted in a bad deformity, if he had not in the beginning of his treatment secured a good position of the limb.

DISCUSSION.

Dr. Gibney had seen a great many cases of hematoma of the sterno-cleido-mastoid muscle, and they invariably got well. He had often wondered whether in some cases of congenital torticollis, actual shortening of the muscle had not been caused by long-continued holding of the head in one position, necessitated by the presence originally of a large hematoma. In these cases of hematoma, there was probably laceration of some of the muscular fibres, with escape of the blood into the sheath, or into the muscular tissue itself.

Dr. N. M. Shaffer said that he had made measurements of the length of the sterno-mastoid muscle in these cases, as well as in normal cases, and his observations showed that there was an arrest of development in the affected muscle, which suggested a possible central lesion, involving the spinal accessory nerve. These cases might arise from traumatism; but unless the destruction of muscular tissue was very great, it would not account for the total arrest of growth.

Dr. Ketch thought that the existence of some deformity in Dr. Phelps's case after such extensive division of the muscles, showed the fallacy of depending altogether upon dividing muscles for the rectification of the deformity of hip disease. As long as the bone disease was active, and muscular spasm was present, deformity would return from this spasm, even after division of the muscles.

Dr. Shaffer also thought that division of the muscles offered only a temporary relief. He had frequently seen recurrence of the deformity after such a procedure in disease of various joints, and especially in cases of tetanoid paraplegia. An examination under ether would determine the amount of muscular resistance; and the breaking up of the intracapsular and extracapsular adhesions, together with subsequent maintenance of the straight position, where all that could be expected in the way of preventing ultimate deformity.

Dr. R. H. Sayre said that much less traumatism was inflicted by dividing the muscles first, rather than by trying to reduce the deformity with the muscles in a state of tension. Dr. Ketch's remarks simply emphasized the importance of proper mechanical treatment after division of the muscles.

Dr. Ketch said that if the reduction of the deformity could be accomplished effectually by mechanical treatment alone, he did not see the advantage of the operation. In answer to a question from Dr. Phelps as to what he would do with a deformity which had not yielded after one year's treatment by traction, he said that such a deformity was probably due to intra-articular changes, and was independent of the muscles; and he would therefore prefer exsection, or other bone operation.

Dr. John Ridlon wished to join the ranks of those who believed in rapid reduction of the deformity—slow reduction caused needless traumatism. In some cases the deformity could be rapidly reduced by mechanical means and without anesthesia; others required anesthesia; and still others were not reducible even then. In this latter class, the first indication was a division of the soft parts, and the second was to maintain the good position until a cure was effected. The average case of flexion through an arc of forty-five degrees, required from twelve to eighteen weeks of treatment with the traction splint for its reduction; and the advocates of the traction splint had just confessed that the deformity would recur after such treatment. The deformity should be overcome in at least a fortnight. Thomas' hip-splint would keep the leg straight, and prevent flexion, adduction, and abduction.

Dr. A. B. Judson had not found that the muscles seriously interfered in the acute stage of the disease, with the reduction of the deformity; and he considered that the reduction could be effected by slow and painless methods, without any harm to the patient. The difficulty in overcoming the deformity was a purely mechanical one, arising from insufficient leverage—only the short distance from the acetabulum to the crest of the ilium.

Dr. Phelps, in closing the discussion on his case, said that the muscles were not divided to overcome reflex muscular spasm, but to overcome deformity; and in obstinate cases of long standing, like the one just presented, this was a safe procedure; while excision of the hip-joint was a serious one. He had not wished to cut the muscles more deeply, and the deformity, although not completely reduced at the time of operation in October, was being constantly diminished by the treatment employed. Statistics showed that a very small percentage of cases treated solely by mechanical means, recovered without deformity; and therefore a resort to operative methods in a certain class of cases, and subsequent mechanical treatment, offered better hopes of success. He would be sorry to cut a tendon and have the case relapse—it would indicate improper treatment. He did not believe in trying to overcome the deformity by Thomas' splint, or any other. During the treatment, in order to get proper leverage, and hold the patient quiet, a long splint was applied to the well leg, extending to the axilla, and the body, limb, and splint enveloped in plaster-of-Paris. No splint could overcome the deformity, or possibly prevent it, which did not pass up on to the thorax. The idea of allowing the patient to walk upon a splint, or upon the diseased limb, was a heresy, which we would eventually renounce. The patient, in his opinion, should use crutches, and he thought that Thomas struck in the right direction; but the splint should be fitted to the patient, and not the patient to the splint. Extension in a line with the axis of the neck of the femur was also necessary to relieve intra-articular pressure by overcoming the contraction of the adductor and abductor muscles.

Dr. JUDSON thought the physiognomy of the case of partial-anchylosis of the jaw was one of arthritis, and the deformity was directly due to the inability to use the jaw, and was not the result of the peculiar shape of the bone. Operation seemed much more successful than the stretching process.

Dr. PHELPS concurred in this opinion, and added that in his experience good results had followed resection of *one* of the tempero-maxillary articulations, as anchylosis was usually found only in one articulation. An incision one and a quarter inches long was made along the zygoma, and the articulation exposed. Chiseling away the articular surface was all that was necessary to cure the case. His cases had presented evidence of arthritis. The anchylosed joint was always on the side of non-development.

Dr. SHAFFER remarked that if he had not heard the history of the case he would have supposed that the patient had had Pott's disease, and had been treated with an apparatus in which the chin-piece had been forced too far backward. He thought there was much rigidity on both sides of the jaw. If the parts relaxed under ether, the evidence would be in favor of arthritis, but if not, it would indicate a permanent contracture, and would demand operation.

Dr. GIBNEY said with reference to the case of posterior deformity of the tibia, that nothing but an osteotomy would correct the deformity. While under ether, the operator should endeavor to bring the fragments nearly into line and then apply retentive apparatus. Subsequently a supra-condyloid osteotomy would be needed. By doing a Macewen's or a Macormac's operation, the subsequent dressings would in great measure correct the antero-lateral curvature. He had frequently seen this occur, sometimes to a marked extent. It was possible that the long rest in bed might have made the bone more yielding.

Dr. SHAFFER thought that the case of arthritis presented by Dr. Ketch answered very well the complete description given by Niemeyer of arthritis deformans. He considered this nothing more than chronic articular rheumatism, and he had seen it both with and without high fever. It closely resembled gonorrheal rheumatism, even in cases where gonorrhea could be absolutely eliminated.

Dr. H. W. Berg said that he had seen a case of gonorrheal rheumatism of the ankle joint, which was quite thoroughly anchylosed and did not recover its function for nearly two years. In such cases the lesion affected chiefly the soft parts, the inflammatory products binding down the tissues so firmly that the joint was virtually anchylosed.

Dr. R. H. Sayre had seen a severe case of arthritis similar to the one presented. After confinement to bed for eight months, suffering from severe pain and high fever, the knee-joint seemed to be absolutely anchylosed, and the patella immovable; but vigorous and persistent massage had secured, after about one year, pretty fair movement. The fact that the joint in the case presented was a little tender, was in favor of the anchylosis not being complete, for the tenderness arose from the pain caused by an almost imperceptible motion of the joint. Persistent and careful efforts at moving the knee-joint, not sufficient to cause pain lasting many hours, would probably give the patient a movable joint.

Dr. Judson called attention to the admirable position of the limb, remarking that a perfectly straight limb was much more stable than one bent at ever so slight an angle. These cases of stiff knee should wear a "lift" on the shoe of the well side, to enable the stiff knee to readily swing past the other, and so avoid awkward tilting of the pelvis at each step.

Dr. Phelps thought that in Dr. Ketch's case there was fibrous anchylosis, and that by breaking this up motion could be secured. In one such case, while forcibly reducing the deformity, the femur was fractured without the exercise of much force, and he called attention to this, because after prolonged rest in one position the bone frequently underwent fatty degeneration, sometimes only a shell of bone remaining. Union of the fracture in his case took place normally. He did not think there was much danger of exciting inflammation by forcible manipulation in these cases, unless the joint had previously been purulent.

Dr. Shaffer's experience had led him to believe that there was considerable danger of exciting inflammation by such treatment, and he would prefer a stiff joint in good position to incurring such risks.

Dr. Ketch, in closing the discussion, said that he believed his case belonged to a class which had never been accurately described. In ordinary cases of arthritis deformans there was involvement of other joints. This was not true of his case, and the sudden onset of such acute symptoms, and the speedy occurrence of anchylosis, were certainly unique. His patient had far too useful a limb to make him desire to incur any risks by employing forcible manipulation.

